# Biomedical Applications of Fermenticin HV6b Isolated from *Lactobacillus fermentum* HV6b MTCC10770

**DOI:** 10.1155/2013/168438

**Published:** 2013-07-29

**Authors:** Baljinder Kaur, Praveen P. Balgir, Bharti Mittu, Balvir Kumar, Neena Garg

**Affiliations:** Department of Biotechnology, Punjabi University, Patiala, Punjab 147002, India

## Abstract

Fermenticin HV6b is a class IIa antimicrobial peptide produced by *Lactobacillus fermentum* HV6b MTCC 10770 isolated from human vaginal ecosystem. It shows growth inhibition of a wide range of opportunistic pathogens of humans, for example, *Bacteroides*, *Gardnerella vaginalis*, *Mobiluncus*, *Staphylococci,* and *Streptococci,* associated with bacterial vaginosis in humans. It does possess an impressive sperm immobilization and spermicidal activity tested against human sperms which makes it an attractive proposition for formulating antibacterial vaginosis and contraceptive products. Apart from this, *in vitro* studies conducted against four different tissue models have indicated its potential to be used as a component of anticancerous drug therapy as it is reported to induce apoptosis in cancerous cells. This information could be integrated in future studies focusing on *in vivo* assessment of anticancerous activity of lactic acid bacterial toxins or bacteriocins.

## 1. Introduction

Bacterial vaginosis (BV) is diseased vaginal state where natural balance of vaginal microflora gets disturbed due to growth of anaerobic bacteria such as *Bacteroides* spp., *Escherichia coli, G. vaginalis, Mobiluncus* spp., *Mycoplasma hominis*, *Peptostreptococcus* spp., *Staphylococci, Streptococci*, and/or viruses [1]. Disease is characterized by a milky or gray vaginal discharge with foul odor, presence of “clue cells,” and an increased vaginal pH of >4.5 [2, 3]. Bacterial vaginosis can induce complications in normal pregnancy [4] and also shows inflammation in pelvic regions. Toxins released by BV pathogens cross placenta and cause permanent brain damage that results in development of neurodegenerative disorders such as Parkinson's disease and Schizophrenia in new borne [5]. Disease also raises risk of sexually transmitted diseases (STDs) including HIV/AIDs, chlamydis, herpes, gonorrhea, and trichomoniasis. Presently, antibiotics such as metronidazole used to treat BV give initial cure rates of approximately 90% or better [6]. It undergoes oxidative metabolism in the liver, which results in formation of several metabolites and becomes widely distributed in the body [7]. Several side effects were observed to be associated with metronidazole antibiotic therapy such as suppression of healthy microflora of vagina, diarrhea, dizziness, headache, loss of appetite, nausea or vomiting, stomach pain, or cramps [8, 9]. Therefore, it is necessary to have an alternative form of treatment that could help in complete eradication of BV. 

Bacteriocins produced by LAB have potential biomedical applications where they may provide valuable alternatives to antibiotics for the treatment of human and animal infections. Antimicrobially active lactobacilli were commonly used to develop products for prevention and treatment of genital infections [10–12]. Bacteriocins that are active against vaginal pathogens are also reported as having spermicidal activity [13, 14]. This feature makes them attractive for formulation of feminine health care and contraceptive products. A number of studies claim that bacteriocins are effective in the prevention of tooth decay and gingivitis and therefore could be included in mouth washes [15–19]. Bacteriocins have also been suggested as a potent antineoplastic agent. They have shown distinct promise as a diagnostic agent for some cancers; bacteriocins have also tested as AIDS drugs [20] but have not progressed beyond *in vitro* tests on cell lines. Fermenticin HV6b is an antimicrobial peptide produced by recently isolated and characterized *Lactobacillus fermentum* HV6b MTCC10770 from human vaginal ecosystem. Bacteriocin is proteinaceous in nature which possesses antimicrobial activity against bacterial vaginosis associated pathogens such as *Bacteroides* species, *Candida albicans*, *Gardnerella vaginalis, Listeria monocytogenes, Micrococcus flavus, Neisseria mucosa, Pediococcus acidilactici, Proteus mirabilis, *and* Staphylococcus albus* [21]. Keeping in view, the health benefits extended by GRAS bacteriocin of lactic acid bacteria, present investigation was carried out with the aim to explore potential biomedical applications of fermenticin HV6b *in vitro.*

## 2. Materials and Methods

### 2.1. Procurement and Maintenance of Culture

*Lactobacillus fermentum* HV6b MTCC10770 was grown in MRS medium, pH 6.5 at 37°C. Standard indicator strains, namely, *Enterococcus faecalis* and *Pediococcus acidilactici* LB42, were procured from Professor R. K. Malik (NDRI, Karnal, India) and cultivated in MRS medium at 37°C. *G. vaginalis* ATCC 14018 was revived and maintained in Casman's medium containing *Gardnerella *active supplement (constituting gentamycin sulphate, nalidixic acid, and amphotericin B) and 5% w/v defibrinated human blood [21]. Bacterial strains were maintained as 20% w/v glycerol stocks stored at −20°C.

### 2.2. Production and Purification of Fermenticin HV6b

Pure bacteriocin preparation was prepared from an overnight grown culture of *Lactobacillus fermentum *HV6b MTCC 10770 in MRS (supplemented with 0.1% w/v Tween 80, pH 6.5) by conventional adsorption-desorption method [22]. 

### 2.3. Bacteriocin Activity Assay

The antimicrobial activity of the bacteriocin preparation was tested against an array of opportunistic human pathogens (as indicated afterwards) using well-diffusion assay as described by Cintas et al. [23]. Bacteriocin activity was calculated as arbitrary unit (AU) and expressed as AU/mL according to standard protocol of Pucci et al. [24].

### 2.4. Determination of Antimicrobial Activity of Bacteriocins

Antimicrobial activity of bacteriocin fermenticin HV6b (crude as well as purified) was characterized using well-diffusion method [23] against *Bacteroides fragilis* MTCC1045, MTCC3298, and MTCC1350, *Candida albicans* ATCC10231 and MTCC183, *Gardnerella vaginalis* ATCC14018, *Micrococcus flavus* ATCC10240, *Neisseria gonorrhoeae* ATCC19424, *N. mucosa* MTCC1772, *Proteus mirabilis* NCIM2387, *Staphylococcus albus* ATCC11631, *S. aureus* MTCC737 and NCTC7447, *Streptococcus agalactiae* NCIM2401, *S. faecalis* MTCC459, *S. pyogenes* NCTC10869, and *S. thermophilus* MTCC1928. General human pathogens used are *Bacillus subtilis* ATCC6633, *Clostridium perfringens* MTCC450, *Escherichia coli* BL21 (DE3) MTCC1679, MTCC1652, and MTCC1650, *Enterococcus faecalis* (Laboratory isolate), ATCC29212, *Klebsiella pneumoniae* NCIM2883 and NCIM2401, *Leuconostoc mesenteroides* MTCC107, *Listeria monocytogenes* MTCC657, *Pseudomonas aeruginosa* ATCC10662, *Salmonella typhi* NCTC5760, *Vibrio cholera* ATCC14104, and *Yersinia enterocolitica* MTCC861. Nonpathogenic microorganisms assayed in the study are *Lactobacillus brevis* MTCC1750, *L. bulgaricus* NCDC253, *L. casei *NCIM2651, *L. helveticus* NCIM2126, *L. leichmanni* NCIM2027, *L. pentosus* NCIM2669, *L. plantarum* NCIM2912, *Lactococcus lactis* subsp. *cremoris* MTCC1484, and *Pediococcus acidilactici* LB42.

### 2.5. Semen Sample Collection and Analysis

Partially purified fermenticin HV6b preparation was used as spermicide to test its effect on motility and immobilization of human spermatozoa. Two semen samples were collected from healthy volunteers in sterile wide-mouth polypropylene containers with a screw cap by self-masturbation on the day of experimentation. Within 1 h of collection, samples were dispensed by mixing a drop of diluted spermicide with a drop of semen and examined under microscope. An approximation is obtained to the highest bacteriocin concentration that does immobilize spermatozoa in 2 min, and the series of dilutions to be used in the test were made in a range below this point. Total sperm count was calculated using a compound microscope (Olympus, 100x) after dilution (1 : 50) of the semen in normal saline. The sperm suspensions were made in small glass tubes, one tube being required for each concentration of the spermicide. The suspensions were made by adding 0.5 mL of semen to each tube of saline or spermicide solution. Samples were placed in an incubator at 37°C for 15 to 30 min to reach that temperature. The percent sperm motility was determined by the progressive (forward) and nonprogressive (vibrating and zig-zag) movement of sperm observed in a compound microscope. The sperm count was calculated using Neubauer haemocytometer from a count of 100–200 sperms using randomly selected (100x) [14].

### 2.6. Treatment of Spermatozoa with Fermenticin HV6b

 Standard protocol of Sutyak et al. [13] was used to determine the effect of purified bacteriocin fermenticin HV6b on the motility of human spermatozoa with little modifications, to measure the effect of fermenticin HV6b on sperm mobility and aggregation after 30 sec exposure time to different concentrations of bacteriocin ranging from 50 to 200 *μ*g/mL of diluted semen sample. The motilities of human spermatozoa cells from random high magnification fields (100x) of the sample were determined in duplicate using atomic force microscope. Results were evaluated according to WHO grade system, and motilities of sperms were divided into four different grades [25]. Grade a sperms have progressive motility. These are the strongest and swim fast in a straight line. Grade b sperms exhibit forward movement but tend to travel in a curved or crooked motion. Grade c sperms show nonprogressive motility because they do not move forward despite the fact that they move their tails, and Grade d sperms have immotile sperms that fail to move at all.

### 2.7. Tissue Models for Testing Anticancerous Activity of Fermenticin HV6b

Tissue model, namely, HepG2 a hepatocarcinoma cell line, was procured from NCCS, Pune, India. A perpetual cervical cell lines (Hela ATCC CCL2), a breast carcinoma cell line (MCF7 ATCC-HTB-22) of *Homo sapiens*, a spleen lymphoblast cell line (Sp2/0-Ag14 ATCC-CRL-1581) of *Mus musculus,* and kidney embryonal cell line (HEK-293 CRL-1573) of *Homo sapiens* were gifted by Dr. Sanjog Jain, Niper, Mohali. Tissues were seeded in culture flasks containing DMEM and RPMI-1640 medium with 10% fetal bovine serum and 100 *μ*g/mL penicillin and streptomycin and cultured in a humidified 5% CO_2_ incubator at 37°C. After reaching 80% confluence, cells were passaged and cultured. Spent culture medium was discarded. The cell layer was rinsed with 0.25% (w/v) trypsin 0.53 mM EDTA solution to remove all traces of serum which may contain trypsin inhibitor. 6.0 to 8.0 mL of growth medium was added, and cells were aspirated by gently pipetting. Tissues were exposed to different concentrations of fermenticin HV6b ranging from 20 *μ*g/mL to 500 *μ*g/mL for 4, 24, and 48 hours. For exposure time over 24 h, the tissues were fed with fresh assay media. After the required exposure time, MTT assay was used to determine overall cell viability. Cell counts of tissue models were checked using haemocytometer. IC-50 (half maximal inhibitory concentration) value for fermenticin HV6b against each cell line is calculated which indicates how much bacteriocin is needed to inhibit the biological system (cell lines) by half [26].

### 2.8. MTT Viability Assay

The MTT assay was carried out according to the protocol given by Kumar et al. [27]. The viability of the cells exposed to bacteriocin was measured as a direct proportion of the breakdown of yellow compound tetrazolium to dark blue water insoluble formazan. Only the metabolically active cells can show this reaction which can be solubilized with DMSO and then quantified. The absorbance of formazon directly correlates with the number of viable cancerous cells. T sperms have progressive movement which ishe liquid in the plate wells was combined with the liquid from the tissue. Mixture is then assayed spectrophotometrically at 540 nm using 96-well plate ELISA reader to determine level of tetrazolium degradation.

### 2.9. DNA Fragmentation

DNA fragmentation analysis reveals the ability of fermenticin HV6b to induce apoptosis in cancer cells. It was carried out as per methodology of Kumar et al. [27] where cells (1 × 10^5^) were treated with 1.0 mg/mL fermenticin HV6b for 48 h and then lysed with 250 *μ*L lysis buffer. After incubation at 37°C for 90 min, 200 *μ*g/mL proteinase K and lithium chloride (0.2% w/v) were added and incubated again for 60 min at 50°C. After incubation was over, suspension was centrifuged at 13,000 rpm for 3 min; aqueous phase was transferred to fresh tube containing deproteinizing mixture of phenol, chloroform, and isoamyl alcohol (25 : 24 : 1) and again centrifuged at 3,000 rpm for 3 min. DNA was precipitated from the aqueous phase with 3 volumes of chilled ethanol containing 0.3 M sodium acetate at 4°C. Samples were subjected to electrophoresis in 1% w/v agarose gel using TAE buffer at 50 V and visualised on a UV transilluminator.

### 2.10. Statistical Analysis

Where appropriate, data are expressed as mean values and standard deviations. Student's *t*-test was used for single comparisons. A probability value of *P* < 0.05 was used as the criterion for statistical significance.

## 3. Results

### 3.1. Antimicrobial Spectrum of Fermenticin HV6b

**Figure 1:**
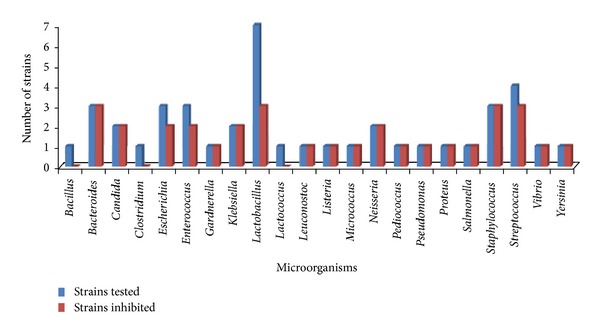
Antimicrobial spectrum of fermenticin HV6b.

Fermenticin HV6b is capable of inhibiting a wide spectrum of human pathogens including *B. fragilis, B. ovatus, B. vulgatus, C. albicans, C. sporogenes, E. coli, E. faecalis, G. vaginalis, K. pneumoniae, L. mesenteroides, L. monocytogenes, M. flavus, N. gonorrhoeae, N. mucosa, P. aeruginosa, P. mirabilis, Staphylococci, Streptococci, S. typhi, and V. cholerae.* It did not show any activity against *B. subtilis, C. perfringens, E. faecalis, L. casei, L. leichmannii, L. plantarum, L. pentosus, L. lactis *subsp.* cremoris, S. agalactiae, *and* Y. enterocolitica.* However, fermenticin HV6b has been reported to exhibit very little growth inhibition of healthy microflora associated with gut and urinary tract as evidenced by a less degree of inhibition in case of *lactobacilli *and *Lactococcus* (Figure 1). Preliminary experiments performed using trypsin and protease-digested, neutralized pure bacteriocin samples led to the idea that inhibitory principle is proteinaceous in nature instead of growth inhibition simply due to acid produced by *L. fermentum* HV6b.

### 3.2. Inhibition of Sperm Motility

**Figure 2:**
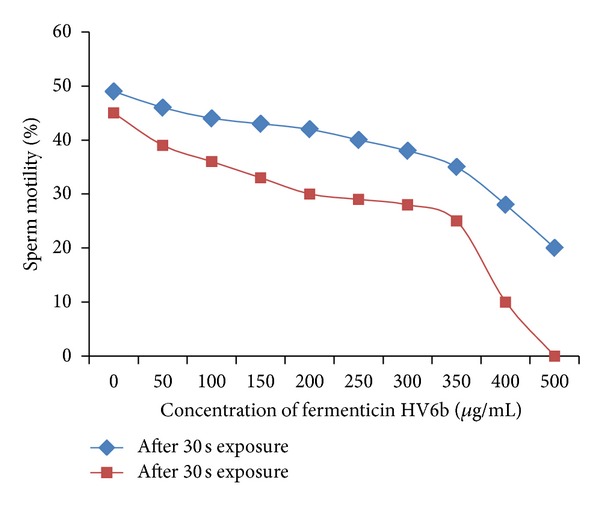
Retardation of sperm motility is a concentration-dependent phenomenon.

**Figure 3:**
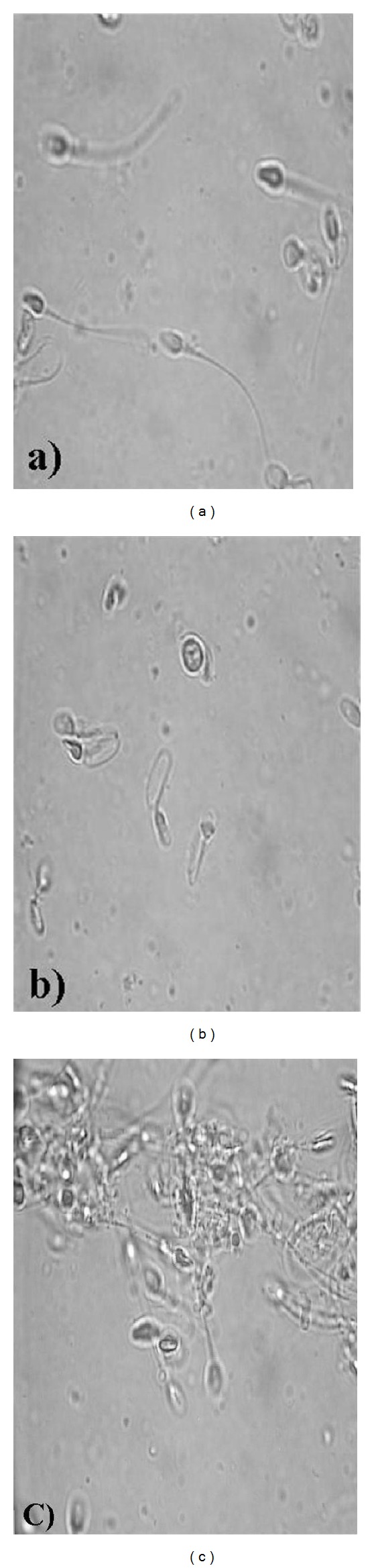
Images showing effect of fermenticin HV6b on human spermatozoa: (a) normal spermatozoa; (b) coiled sperm tails; (c) sperm aggregation and immobilization.

Fermenticin was shown to significantly (*P* < 0.05) reduce motility of the human spermatozoa in a concentration-dependent manner (Figure 2). Normal untreated spermatozoa have progressive movement which is characteristic of “Grade a” category according to WHO. But upon exposure to higher concentrations of the fermenticin HV6b, coiling, clumping, and agglutination of sperms were observed that falls in Grade d. The effect of bacteriocin on the forward progression of human sperms and their aggregation was observed to be a dose-dependent interaction. Sperms show a steady decrease in forward progression (up to 50%) on exposure to increasing concentration of bacteriocin, with all progression halted at the concentration of 200 *μ*g/mL for fermenticin HV6b as compared to control samples. Tails of sperm cells became curved or coiled as result of bacteriocin treatment, indicating their damage beyond a simple restriction of movement (Figure 3). Coiling of the sperm tails is considered to be an abnormality and may indicate damage to the plasma membrane [13, 14]. In a suspension of spermatozoa treated with sublethal concentration of bacteriocin, the speed was reduced, and if such reduction was compared with a control suspension, it was found to be proportional to the concentration of fermenticin in the test suspension. The degree of reduction in speed would be a convenient method of assessing the relative spermicidal power of purified bacteriocins (Table 1). The results established fermenticin HV6b as a general spermicidal agent.

### 3.3. Anticancerous Activity of Fermenticin HV6b

**Figure 4:**
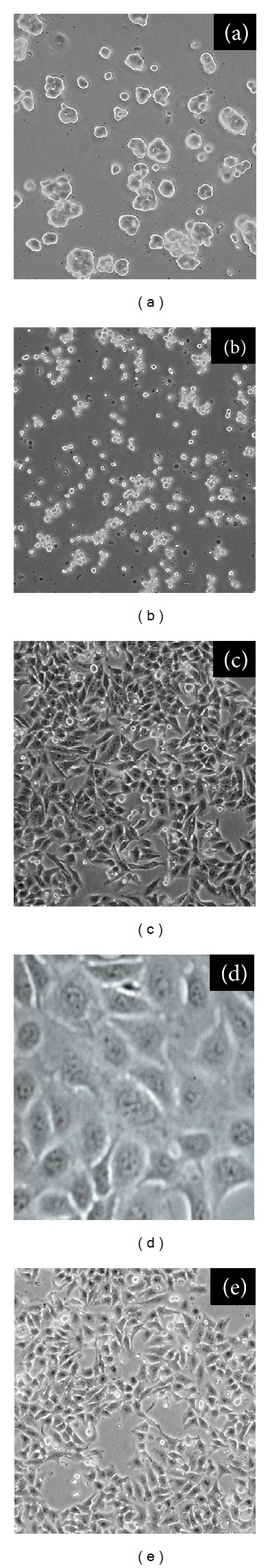
Morphological properties of cancerous cell lines used in the study: (a) MCF-7; (b) Sp2/0; (c) Hep G2; (d) HEK-293; (e) HeLa.

Cytotoxicity of fermenticin HV6b was assessed on several cancerous cell lines with different morphologies and physiology (Table 2). Figure 4 shows cell viability after incubation for 24 h in a medium containing fermenticin HV6b. MCF-7 and HEK-293 presented a slight sensitivity to this bacteriocin, whereas HeLa, Sp2/O, and Hep G2 cell lines were sensitive at different degrees to the fermenticin HV6b toxic effect. After 24–48 hours of exposure to the fermenticin HV6b, the epithelial tissue models retained only a low level of viability. Total cell viability drops below 50% at any point due to toxicity of the antimicrobial factor. The cell viability of MCF-7 cell was 46% whereas that of HeLa cells was 25%. Sp2/0 cells, HEK-293 cells, and HepG2 cells showed 30%, 38%, and 20% cell viability after treatment with 1 *μ*g/mL fermenticin, respectively. Results showed that fermenticin HV6b is effective against cancerous cell lines, and, therefore, it is suggested to be used for clinical purposes. IC-50 values have been calculated for cytotoxic assay which indicates that 0.9 *μ*g/mL of fermenticin HV6b was required to inhibit the growth of MCF 7 by 50%, whereas IC-50 value of bacteriocin to inhibit HEP G2 was found to be as less as 0.1 *μ*g/mL. Figure 4 shows the modifications in the different cell lines with the treatment of fermenticin HV6b.

### 3.4. DNA Fragmentation

**Figure 5:**
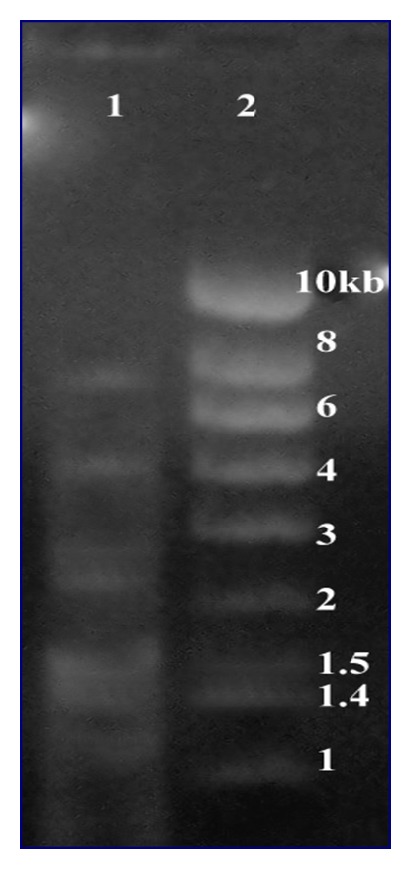
Analysis of genomic DNA on 1% agarose gel. Lane 1: fragmented genomic DNA of bacteriocin treated cells. Lane 2: 0.05 to 10 Kb DNA ruler (Novagen).

Apoptosis in tissues was examined by DNA fragmentation assay. Cells were treated with fermenticin HV6b at 1.0 mg/mL for 48 h. Fermenticin HV6b was reported to increase DNA fragmentation (Figure 5) in cancerous cells. These results provided evidence that fermenticin HV6b induces cell-cycle arrest and apoptosis in cells. 

## 4. Discussion

Human lactobacilli are used as probiotics to restore and maintain a healthy urogenital tract as an alternative to conformist chemotherapy. Present study therefore aimed at investigating *in vitro* efficacy of fermenticin HV6b in inhibiting growth of BV associated and other human pathogens and exploring spermicidal and anticancerous activities so that efficacy of *L. fermentum* HV6b could be established as a general human probiotic agent. Lactobacilli have been recommended as GRAS biotherapeutic agents for cure as well as prevention of human gastrointestinal and vaginal diseases. Colonization of the infected tissue by health promoting LAB particularly prevents infection by synthesizing a variety of antagonistic factors such as bacteriocins, diacetyl, H_2_O_2_, and fungicidal agents, by competing for available nutrients and mannose sugar, and by interfering pathogen attachment to cell surface receptors. An acidic pH of vagina alone is not sufficient to inhibit vaginal pathogens and to prevent bacterial vaginosis [14]. Thus, bacteriocin based therapeutics are urgently desired to cure such diseases and to overcome problems associated with antibiotic therapy such as diarrhea, poor compliance, and recurrence of vaginal infections. There is increasing body of evidence that indicates potential of GRAS lactic acid bacteria in maintaining and restoring gut homeostatis [28]. Use of living probiotic culture may have prophylactic applications, but use of purified bacteriocins appears to be more attractive for eradicating an established infection [29].

Several investigators have isolated and partially purified bacteriocin from different species of lactobacilli [30]. Most of these studies were carried on nonhuman strains which were predominantly isolated from food. In a study, inhibition of urinary tract infections (UTI) pathogens such as *E. faecalis*, *E. faecium,* and *N. gonorrhoeae* was reported by bacteriocin vaginal *Lactobacillus salivarius* strain [21]. Similarly, another study reported killing of a wide range of Gram-positive and Gram-negative pathogenic bacteria by bacteriocin HV219 of *L. lactis* origin which itself was isolated from human vagina [30].

Herein, we report that fermenticin HV6b produced by *L. fermentum* HV6b could target vaginal pathogens while leaving the healthy vaginal microflora intact as evidenced in the present study by performing growth inhibition assays on both human normal (*L. brevis* MTCC1750, *L. bulgaricus* NCDC253, *L. casei *NCIM2651, *L. helveticus* NCIM2126, *L. leichmannii* NCIM2027, *L. pentosus* NCIM2669, *L. plantarum* NCIM2912, *Lactococcus lactis* subsp. *cremoris* MTCC1484, and *Pediococcus acidilactici* LB42) as well as pathogenic gut flora including *B. fragilis, B. ovatus, B. vulgatus, C. albicans, C. sporogenes*, *E. coli, E. faecalis, L. monocytogenes, M. flavus, N. gonorrhoeae, N. mucosa, P. aeruginosa, P. mirabilis, Staphylococci, Streptococci, S. typhi, *and* V. cholera*. It is of interest to the food and pharmaceutical industries both as it exhibits such as broad inhibitory spectrum against food-borne pathogens, spoilage organism, and human opportunistic pathogens of gut and urinary tract. Microorganisms such as *S. pyogenes *causing superficial skin infections to life-threatening systemic diseases and *L. monocytogenes* that causes spectic abortion, newborn, and adult septicemia, listeriosis, meningitis, and meningoencephalitis in immune-deficient persons [31] are also susceptible to fermenticin. In accordance with earlier reports, we herein report the utility of fermenticin to control human diseases as the best alternative to antibiotic therapy as it could be safely incorporated into personal care applications aimed at treatment of bacterial vaginosis [32, 33]. 

Results of present study also establishes fermenticin as a general spermicidal agent. Previous studies on subtilosin [13], nisin [34], and pediocin CP2 [14] have already reported them as potent spermicidal agents. Currently, used anticancer drugs have been shown to induce apoptosis in susceptible cells. Apoptosis is an important process of many pathological conditions. A series of studies have provided convincing evidence, suggesting that bacteriocin induces apoptosis of vascular endothelial cells. Principle of apoptosis was described by Vogt [35] which shows it as a programmed death of cells, which may even occur in multicellular organisms. Various biochemical changes such as loss of cell membrane asymmetry and attachment, cell shrinkage, nuclear fragmentation, chromatin condensation, and chromosomal DNA fragmentation take place during apoptosis. DNA fragmentation occurs at an end stage of apoptosis, which includes activation of calcium and magnesium dependent nucleases that degrade genomic DNA. The results presented here indicate cytotoxic effect of fermenticin HV6b on various cancerous cell lines. The cytotoxic effect on cancerous cells from human origin was also reported earlier [27, 36]. The uniqueness of the bacteriocins lies in their interaction with the cell surface without penetrating the target cells, yet affecting cell division and DNA synthesis [37]. Bacteriocins are highly specific in their membrane interaction which is related to the unique receptors found in different bacterial species or types [38].

## 5. Conclusion

Class IIa bacteriocins have ability to target a relatively wide range of pathogenic bacteria. This is an important attribute of GRAS bacteria that could be exploited to replace antibiotic therapy for treating bacterial vaginosis gut infections and peptic ulcers. Adhesive capacity and colonization of the gastrointestinal tract of humans and animals by probiotic lactobacilli including *L. fermentum* have been extensively investigated [39–41]. Organism can be delivered at the site of action, that is, gut or vagina, in the lyophilized capsular form where it can multiply and establish itself. Organism has a property to synthesize bacteriocin which can further help the bacteria to eradicate pathogenic organism from the inhabited area through competitive exclusion. Several *in vitro* studies have established GRAS bacteriocins as potent spermicidal and anticancerous agents. Preliminary experiments with fermenticin HV6b have shown its potency for formulating personal care products. Continued study is however desired for having complete insight into mechanism of killing sensitive bacterial species. Its cytoxicity has been proved for cancerous cell lines and which is attributed through the induction of programmed cell death or apoptosis. In future, this information could be integrated and exploited to fully explore the suitability of fermenticin HV6b as *in vivo* therapeutics against BV and various forms of cancers.

## Figures and Tables

**Table 1:** Dose-dependent inhibition of sperm motility by fermenticin HV6b.

S. no.	Concentration of bacteriocin (*μ*g/mL)	Mobile spermatozoa after 30 sec of exposure	Mobile spermatozoa after 60 sec of exposure	Motility grade after 1 min of exposure	Motility grade after 10 min of exposure
1	0	49 ± 1	45 ± 1	Grade a	Grade a
2	50	46 ± 1	39 ± 2	Grade a	Grade b
3	100	44 ± 2	37 ± 1	Grade a	Grade b
4	150	43 ± 1	33 ± 2	Grade b	Grade b
5	200	42 ± 1	30 ± 1	Grade b	Grade c
7	250	40 ± 3	29 ± 3	Grade c	Grade c
8	300	38 ± 1	28 ± 2	Grade c	Grade d
9	350	35 ± 2	25 ± 2	Grade d	Grade d
10	400	28 ± 2	10 ± 1	Grade d	Grade d
11	500	20 ± 3	0 ± 1	Grade d	Grade d

Each data is mean ± standard deviation; *P* value < 0.05; *F* crit (4.4138) < *F* value (14.5832).

**Table 2:** Cytotoxic effect of fermenticin on cancerous cells.

Cancer cell lines tested	% cell viability at bacteriocin concentration			IC-50 value (*μ*g/mL)
	0.1 *μ*g/mL	1 *μ*g/mL	10 *μ*g/mL	
MCF-7	88 ± 1.9	46 ± 2.1	10 ± 1.7	0.9 ± 0.016
Sp2/0	70 ± 1.4	30 ± 1.7	9 ± 1.5	0.5 ± 0.012
Hep-G2	50 ± 1.8	20 ± 1.5	10 ± 1.9	0.1 ± 0.014
HEK-293	78 ± 2.4	38 ± 2.8	8 ± 2.6	0.6 ± 0.015
HeLa	65 ± 1.3	25 ± 1.2	6 ± 1.6	0.4 ± 0.013

Each data is mean ± standard deviation; *P* value < 0.05; *F* crit (3.2388) < *F* value (62.3008).
